# Getting to grips with circular chromosomes

**DOI:** 10.7554/eLife.60150

**Published:** 2020-08-05

**Authors:** Constance Nugent, Katsunori Sugimoto

**Affiliations:** 1 Department of Molecular, Cellular and Systems Biology, University of California, RiversideRiversideUnited States; 2 Department of Microbiology, Biochemistry and Molecular Genetics, Rutgers New Jersey Medical SchoolNewarkUnited States

**Keywords:** single chromosome yeast, CST complex, chromosome end fusion, telomere protection, homologous recombination, telomeres, *S. cerevisiae*

## Abstract

A strain of budding yeast that contains one large chromosome reveals how the telomere capping complex CST maintains linear but not circular chromosomes.

**Related research article** Wu ZJ, Liu JC, Man X, Gu X, Li TY, Cai C, He MH, Shao Y, Lu N, Xue X, Qin Z, Zhou JQ. 2020. Cdc13 is predominant over Stn1 and Ten1 in preventing chromosome end fusions. *eLife* **9**:e53144. doi: 10.7554/eLife.53144

Genetic material is stored inside cells in structures called chromosomes, which have a repetitive sequence known as a telomere at each end. Specialized proteins bind to these sequences to form a protective 'cap' that protects the chromosome and prevents it from fusing with other chromosomes. The enzyme telomerase also helps maintain chromosomes by adding repetitive sequences of DNA to the ends of telomeres.

One of the most widely studied capping molecules is a protein called Cdc13 that binds to certain types of single-stranded DNA in budding yeast, and forms a complex with two other proteins (Stn1 and Ten1) that recruits telomerase (Wellinger and Zakian, 2012). Several lines of evidence suggest that this CST complex also recruits a DNA replication enzyme called primase-Polα, and can regulate the activity of this enzyme at the ends of chromosomes as well as other locations in the genome (Giraud-Panis et al., 2010; Price et al., 2010; Barbero Barcenilla and Shippen, 2019).

Similar complexes have also been identified in other eukaryotes, including mammals, which contain Stn1, Ten1, and another protein called CTC1 within their CST complex (Giraud-Panis et al., 2010; Price et al., 2010). However, it has proved challenging to work out the roles performed by the different proteins in the CST complex because cells that lack just one of these proteins struggle to survive (Figure 1A). Now, in eLife, Jin-Qiu Zhou and co-workers at the Chinese Academy of Sciences and ShanghaiTech University – including Zhi-Jing Wu as first author – report the results of experiments that help improve our understanding of the CST complex (Wu et al., 2020).

**Figure 1.**
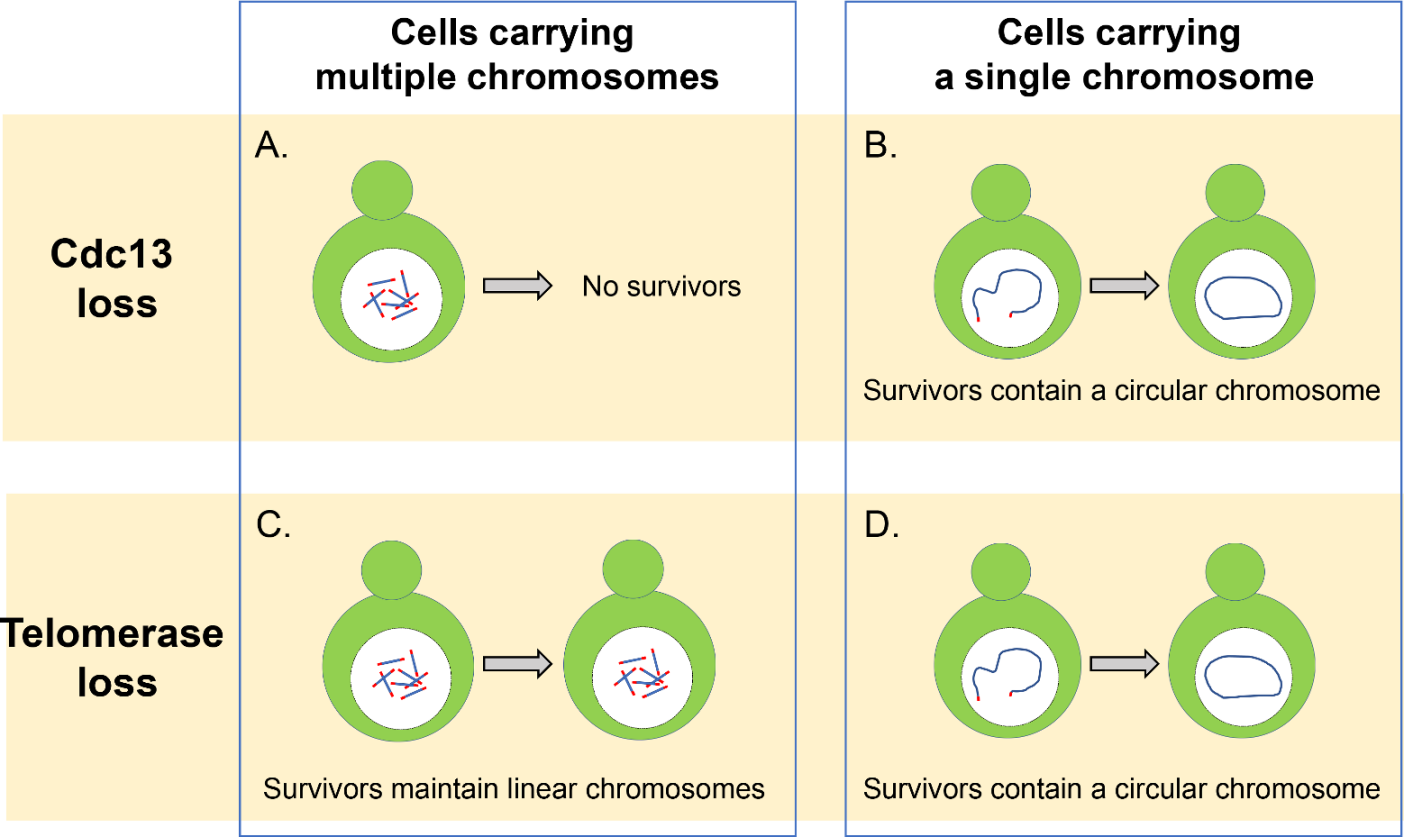
Cdc13 and telomerase are essential for maintaining linear chromosomes. (**A**) Yeast cells with multiple linear chromosomes require the capping protein Cdc13 to protect their telomeres and prevent chromosomes from fusing. Without this protein these cells cannot survive. (**B**) Cells that have a single linear chromosome can survive without Cdc13 by fusing the ends of their chromosome together to form a circular ring. (**C**) In the absence of the enzyme telomerase, cells with multiple linear chromosomes are able to survive by employing DNA recombination pathways which can amplify the telomere sequence or the DNA segments that sit between the chromatin and telomere sequence. (**D**) Cells with a single linear chromosome survive the loss of telomerase by fusing together to form a circular chromosome using homologous recombination, similar to what happens in cells lacking the protein Cdc13.

Image credit: Constance Nugent and Katsunori Sugimoto.

First, Wu et al. investigated how deleting the CST complex affected the viability of a strain of budding yeast in which all of its 16 chromosomes were fused together to form a single circular chromosome (Shao et al., 2019). They found that removing CST did not stop the cells from proliferating or lead to more cell deaths, even when the circular chromosome contained the repetitive telomere sequences. It appears, therefore, that the main role of the CST complex is to maintain linear chromosomes and to prevent chromosomes from fusing with other chromosomes, and that it is not essential for the replication of internal telomere sequences.

In addition to forming a circular ring, the 16 chromosomes of budding yeast can also be fused together to form a single linear chromosome (Shao et al., 2018). Wu et al. found that removing the CST complex greatly reduced the viability of these cells, but some of these cells were able to survive by fusing the ends of their linear chromosome to form a circular ring (Figure 1B). Individually deleting the genes that code for the different proteins of the CST complex revealed that cells lacking Cdc13 displayed a higher rate of fusion than cells missing the genes for Stn1 and Ten1. This suggests that Cdc13 plays a dominant role in inhibiting the fusion of chromosomes, and that Stn1 and Ten1 contribute to the protection of telomeres independently of Cdc13. However, the details of this mechanism still remain unclear and require further investigation.

In wild-type cells that contain multiple chromosomes, it is rare to find fused or circular chromosomes, even when the activity of telomerase has been compromised: this is because cells can extend and maintain telomeres by using a mechanism called homology-directed recombination that repairs double stranded breaks in DNA (Figure 1C). However, Wu et al. found that reducing the number of chromosomes led to more fusions being detected in cells lacking the enzyme telomerase. This suggests that reducing the number of chromosomes increases the likelihood that cells will be able to produce circularized chromosomes and survive the loss of telomerase.

It was thought that fusing the two ends of the singular linear chromosome would rely on a DNA repair pathway called the nonhomologous end-joining (NHEJ) pathway (Haber, 2016). However, Wu et al. demonstrated that in the absence of telomerase, chromosome fusion depended on Rad52, which plays a critical role in the homologous recombination of DNA breaks in budding yeast (Figure 1D). It is possible that the cells used in this study rely on the Rad52 pathway for chromosome circularization because the single chromosome has an inverted telomere sequence near one end of the chromosome. If such a sequence were deleted, cells might undergo end-to-end fusion through the NHEJ pathway that is more common in human cells (Palm and de Lange, 2008). Additional experiments showed that this finding was not due to a loss in NHEJ activity and that this pathway is able to fuse linearized plasmids in budding yeast cells.

The work of Wu et al. provides new insights into how chromosomes fuse together and how telomeres are maintained independently from the telomerase enzyme. Moreover, the findings from this study might go beyond yeast and improve our understanding of various human medical syndromes caused by the ends of chromosomes fusing to form ring shapes (Pristyazhnyuk and Menzorov, 2018).
